# Advancing the Translational Pipeline: From In Vivo and In Vitro 2D Models to Next-Generation Cell Culture Models for Amyotrophic Lateral Sclerosis Modelling and Drug Discovery

**DOI:** 10.3390/biomedicines14102333

**Published:** 2026-10-14

**Authors:** Elisabetta Carata, Stefania Mariano, Bernardetta Anna Tenuzzo, Elisa Panzarini

**Affiliations:** 1 Department of Biological and Environmental Sciences and Technologies (Di.S.Te.B.A.), University of Salento, 73100 Lecce, Italy; elisabetta.carata@unisalento.it (E.C.); bernardetta.tenuzzo@unisalento.it (B.A.T.); 2 CEDAD (Centre of Applied Physics, Dating and Diagnostics), Department of Mathematics and Physics, University of Salento, 73100 Lecce, Italy; stefania.mariano@unisalento.it

**Keywords:** amyotrophic lateral sclerosis (ALS), brain organoids, organ-on-a-chip, induced pluripotent stem cells (iPSCs), assembloids, translational medicine, disease modelling, gene editing, 3D bioprinting

## Abstract

**Background**: Amyotrophic lateral sclerosis (ALS) is still a global health challenge, as there is no effective treatment due to the inadequacy of conventional experimental platforms for comprehending pathogenic mechanisms and developing therapy. **Objectives**: This review details the recent progress and clinical utility of next-generation iPSC-derived 3D in vitro systems integrated with microfluidics, CRISPR-Cas9 gene editing, multi-omics readouts, and AI-based machine learning, which are emerging as transformative platforms for recapitulating key ALS pathological hallmarks. **Methods**: A literature search was performed using PubMed and Web of Science, including only English-language publications. All the published papers collected covered the period from January 2015 to June 2026. **Results**: Human induced pluripotent stem cell (iPSC) cerebral organoids, assembloids, and organ-on-a-chip technologies are revolutionizing research into ALS by providing a way to model the complex spatial organization, cellular heterogeneity, and neural network dynamics of the human brain. Moreover, recent bioengineering advancements enable the inclusion of functional neurovascular units, the integration of resident immune cells (microglia), and the use of 3D bioprinting to accelerate the functional maturation of age-related phenotypes. CRISPR-Cas9 genome editing, OMICS, and machine learning improve tools for studying ALS and identifying therapeutic targets. **Conclusions**: Novel cell culture models may bridge the gap between preclinical animal models and human clinical trials and reduce the duration and costs of translation. Future work is likely to integrate bioprinting with organ-on-chip microfluidics, CRISPR-based genetic editing, and multi-omics readouts to build increasingly faithful, patient-specific ALS models for both basic research and precision therapeutics.

## 1. Introduction

Amyotrophic lateral sclerosis (ALS) is a fatal neurodegenerative syndrome characterized by an impairment of motor system functionality upon the degeneration of the upper and lower motor neurons (MNs) in the brain and spinal cord, leading to skeletal muscle failure. The mean age of onset ranges between 54 and 67 years, and the median survival period following the onset is 2–5 years; it is slightly more common in men than in women. Several regions of the central nervous system (CNS) as well as other body systems are affected in ALS and cause the neuropathological spectrum of ALS, including progressive muscular atrophy, swallowing and speech complications, cognitive abnormalities, autonomic nervous system impairment, weight loss and hypermetabolism, and abnormal eye movement [1,2,3].

ALS was first described in 1869, and today it represents the most common MN disease in adulthood. Global projections indicate that ALS cases could significantly rise over the coming decades due to ageing populations, and it has been estimated that the number of ALS patients will increase by 20% in Europe and by 34% in the USA from 2015 to 2040, suggesting that it is not correct to consider it a rare disease [4].

Two forms of ALS are known, familial (fALS) and sporadic (sALS); the first includes about 10–15% of ALS cases, and the second the remaining 85–90%. In 50% of fALS cases and in at least 5% of sALS cases, mutations occur in Superoxide dismutase-1 (SOD1), chromosome 9 open reading frame 72 (C9ORF72), TAR DNA binding protein 43 kDa (TARDBP, encoding TDP-43), and fused in sarcoma (FUS) genes. SOD1 mutations can occur at any level of gene sequence and determine a toxic gain-of-function pathology; the presence of hexanucleotide repeat expansions (HREs) in the C9ORF72 gene induces loss of function of the C9ORF72 protein and toxic gain of function; mutation of the TARDBP gene causes the formation of TDP-43 aggregates; and mutations in the FUS gene cause a pathological cytoplasmic redistribution of the encoded protein with consequent failure to translocate to the nucleus. Recently, a Genome-Wide Association Study (GWAS) and Whole-Exome Sequencing (WES) identified 12 novel genes (ANXA11, ARPP21, CAV1, C21ORF2, CCNF, DNAJC7, GLT8D1, KIF5A, NEK1, SPTLC1, TIA1, and WDR7) whose mutations affect protein homeostasis, DNA repair, RNA metabolism, vesicle transport, and mitochondrial function in ALS pathogenesis, representing a risk factor through interplay with environmental factors [5]. In fact, there are environmental factors, including exposure to toxins such as lead and smoking, that are thought to place people at higher risk of developing the disease. Moreover, it has been noted that individuals who have served in the military and professional soccer and football players have a higher risk of developing ALS, although it is unclear exactly why this occurs [6].

To date, ALS pathophysiology is still not deeply understood, and, consequently, effective pharmacological treatments are not available. In addition to this, diagnosis is also hard since predictive biomarkers are lacking. Alterations in DNA repair, RNA metabolism, mitochondria, and cytoskeletal trafficking are the most recurrent molecular ALS features, and, except for SOD1-ALS cases, the presence of TDP-43 cytoplasmic inclusions in neurons and glia, impairing microtubule stability and MN growth and function, represents an almost universal biochemical signature [7]. Diagnosis is based only on clinical assessment via nerve conduction monitoring and electromyography, while genetic analysis confirms and categorizes ALS. Finally, no pharmaceutical or nonpharmaceutical intervention stops the progression of the disease or prolongs the survival of ALS patients, and treatment aims only to improve the quality of life of patients by mitigating the symptoms (pain, muscle cramps, dysphagia, dementia, and respiratory insufficiency); patient care includes a multidisciplinary approach.

Over 60 drugs are currently being tested, but only four of them are approved: Riluzole, the first drug approved by the U.S. Food and Drug Administration (FDA) as capable of providing neuroprotection by acting as a glutamate transmission inhibitor; Edaravone, an antioxidant agent approved in Japan in 2015 and by the FDA in 2017; AMX0035, a taurursodiol-sodium phenylbutyrate formulation able to mitigate mitochondrial dysfunction and endoplasmic reticulum stress that is approved by the FDA; and Qualsody (tofersen), which is FDA-approved only for patients with the SOD1 mutation [8]. Currently, 52 ongoing ALS experimental therapies and 66 concluded clinical trials have been reported on (https://www.arisla.org/sla/terapie-sperimentali-nel-mondo-2/, accessed on 25 August 2026). The studies have yielded disappointing results, and the failures are driven by low bioavailability, poor CNS delivery, dosing constraints, a lack of reliable biomarkers and clinical models, delayed diagnoses, and incomplete knowledge of ALS’s pathophysiology. Thus, advanced therapies and early diagnostic tools remain an urgent priority. Beyond cell-autonomous motor neuron vulnerability, ALS is increasingly recognized as a multi-systemic disorder involving non-cell-autonomous toxicity, systemic inflammation, and inter-organ crosstalk across the body’s regions, including the gut, immune system, muscles, and others [9]. This complexity clashes with the lack of experimental models suitable for recapitulating all the physiological or pathological features of ALS accurately. Traditional monolayer cell cultures cannot model ALS’s complex spatial dynamics or multi-lineage 3D architectures. Consequently, capturing the full spectrum of ALS’s pathophysiology requires a deep knowledge of the complex biological mechanisms and, therefore, the availability of bioengineered human platforms capable of integrating multicellular 3D microenvironments with dynamic fluidic flow.

**Figure 1:**
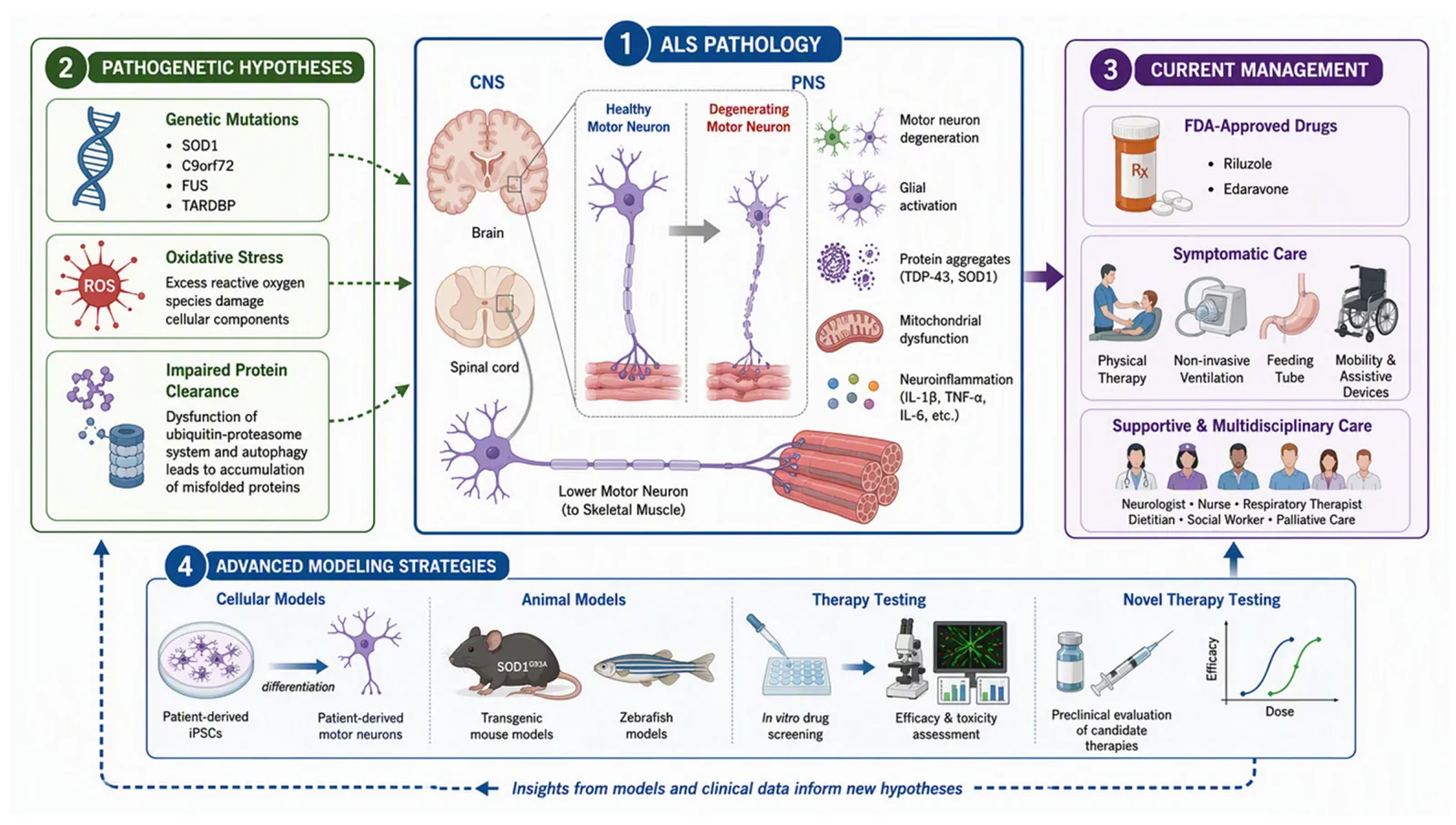
Comprehensive overview of ALS pathogenesis, management, and modelling strategies. Motor neuron degeneration, mitochondrial dysfunction, protein aggregation, neuroinflammation, and glial activation are key drivers of ALS involving genetic mutations (e.g., SOD1, C9orf72), oxidative stress, and impaired protein clearance mechanisms. The clinical management approaches are multi-faceted and combine FDA-approved drugs (Riluzole, Edaravone), symptomatic care (physical therapy, ventilation, mobility aids), and a supportive multidisciplinary team. The advanced modelling strategies critical for future therapeutic developments include patient-derived cell models (iPSC-to-motor neuron differentiation), animal models (transgenic mice, zebrafish), and the subsequent platforms for in vitro drug screening and preclinical evaluation. The figure was generated with the support of FigureLabs AI.

In Figure 1, a comprehensive translational framework of ALS, spanning pathogenetic hypotheses, neuropathology, clinical management, and advanced modelling strategies for drug discovery, is reported.

In this review, after briefly summarizing the main in vivo and in vitro models used in ALS research and their specific limitations, we focus on advanced models, including organoids, assembloids, and organ-on-a-chip technologies representing the most recent systems for ALS modelling. We explore the capability of iPSC-derived 3D systems in recapitulating key ALS pathological hallmarks; highlight how the integration of CRISPR-Cas9 genome editing enables the generation of high-fidelity isogenic controls, reducing experimental variability and facilitating targeted gene correction or knockout strategies; address critical challenges; and outline how the combination of multi-dimensional 3D models with microfluidics, gene editing, multi-omics readouts, and AI-based machine learning will accelerate the precision medicine pipeline and therapeutic discovery for ALS.

## 2. Literature Search and Review Methodology

### 2.1. Literature Search and Study Selection

Articles and reviews regarding amyotrophic lateral sclerosis and models used in the research were identified from the Web of Science and PubMed databases to screen peer-reviewed, English-language publications. Web of Science (WoS, Clarivate Analytics, Philadelphia, PA, USA), one of the most complete and accredited database platforms, contains more than 22,000 peer-reviewed journals. PubMed collects more than 37 million citations for biomedical literature from MEDLINE, including life science journals and books. All of the published papers we collected cover the period from January 2015 to June 2026. In this study, the search terms and Boolean operators utilized were defined as follows: TS = (amyotrophic lateral sclerosis) AND TS = (models OR in vitro studies OR in vivo studies) AND TS = (new generation models) AND TS = (microfluidics) AND TS = (gene editing) AND TS = (OMICS) AND TS = (machine learning). To ensure a structured and transparent selection process, a literature screening flow chart following the PRISMA framework was implemented. Studies were included if they investigated non-human in vivo models, human 2D cell cultures, or next-generation 3D cellular systems (such as brain, spinal cord, or neuromuscular organoids, chimeroids, assembloids, and organ-on-a-chip microfluidic platforms) for ALS pathogenesis, biomarker discovery, or drug screening. Articles were excluded if they were non-English publications, conference abstracts, or studies not directly related to ALS experimental modelling and translational platforms.

### 2.2. Review Design and Metadata Extraction

This review was designed to evaluate the translational trajectory of ALS experimental platforms, tracing the paradigm shift from traditional 2D cell cultures and non-human animal models toward advanced human iPSC-derived 3D bioengineered architectures, CRISPR-Cas9 genome editing, multi-omics profiling, and AI-driven predictive modelling. All retrieved citations were independently screened at both the title/abstract and full-text levels.

Relevant metadata and experimental parameters from the included studies were systematically extracted and synthesized into comparative tables (Section 4, Section 5.4, Section 6 and Section 9). Extracted parameters included author information, publication year, specific model type, genetic background or mutations modelled (e.g., SOD1, C9orf72, TARDBP, FUS), key pathological readouts, and reported translational advantages or technical limitations. To minimize potential selection and reporting biases, all retrieved citations were screened at both title/abstract and full-text levels. The quality of bibliographic references was evaluated by analyzing source reliability, methodological rigour, temporal and thematic relevance to the topic, and authors’ expertise. Moreover, journal Impact Factor and transparency indicators (FAIR open data, conflict of interest disclosures, and study pre-registration) were considered.

### 2.3. Figure Construction and Graphic Protocols

**Figure 2:**
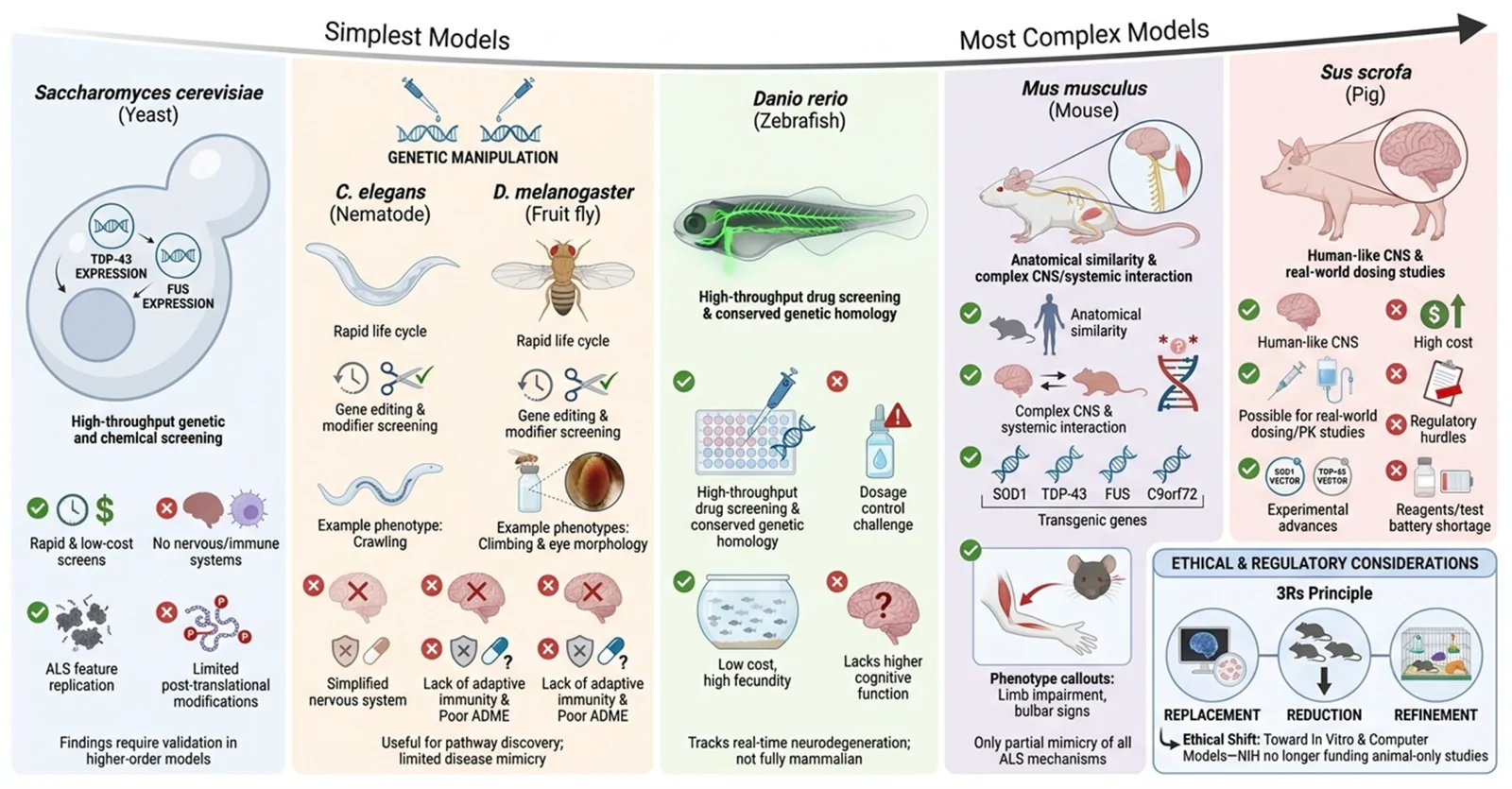
Comparative overview, key strengths, and translational limitations of non-human experimental models in ALS research. The non-human experimental systems are ordered by structural and biological complexity, ranging from unicellular yeast to large mammalian models (Saccharomyces cerevisiae, Caenorhabditis elegans, Drosophila melanogaster, Danio rerio, Mus musculus, and Sus scrofa). Each of them is pivotal for different features of ALS and presents challenges in clinical translation. In addition, ethical limitations are suggested by frameworks governing humane and compliant model organism research. The figure was generated with the support of FigureLabs AI. The asterisks indicate the sites of mutations.

To enhance visual communication and provide clear conceptual frameworks, graphical schematics were conceptualized and constructed using dedicated digital design tools and AI-assisted graphic engines (including FigureLabs AI). Schematics detailing ALS disease management (Figure 1) and non-human model comparisons (Figure 2) were produced. All high-resolution graphics and vector elements were formatted at 600 DPI to meet strict publication and reproducibility standards.

## 3. Non-Human In Vivo Models in ALS Research: Potential and Limits of Their Translatability

Non-human models, including yeast, worms, zebrafish, mice, and pigs, are valuable tools in ALS research due to their accessibility for genetic modification. Unfortunately, they show limitations in drug development studies, as both genetic and epigenetic factors are involved in ALS occurrence and, so, doubts about the translatability of animal disease models to human patients exist (Figure 2).

The yeast *Saccharomyces cerevisiae* is a valuable model for studying ALS because it can express ALS-linked human proteins (like TDP-43 and FUS) and, so, it is possible to screen thousands of genetic and chemical modifiers with respect to the time and cost required for mammalian models, and accurately replicate several pathologic features of ALS, such as protein misfolding and aggregation, mitochondrial dysfunction, DNA damage, and RNA toxicity [10]. Almost 37 human genes capable of suppressing FUS-induced toxicity have been identified using *S. cerevisiae* [11]. Despite the advantages of high-throughput genetic screening, *S. cerevisiae* presents key limitations for investigating intricate ALS mechanisms due primarily to biological differences between single-celled fungi and complex mammalian nervous systems. They lack many mammalian-specific signalling pathways and protein regulatory networks, and their capacity for post-translational modifications, which heavily dictate how human proteins misfold and aggregate, is limited. Moreover, yeasts cannot model the systemic and environmental factors or the complex immune responses characteristic of ALS in humans. So, they are typically used for rapid, initial genetic and chemical screenings, and the findings must always be validated in higher-order animal models, such as *C. elegans*, *D. melanogaster*, zebrafish, or mice.

Particularly useful for identifying pathways involved in protein toxicity and neuronal stress in ALS are two invertebrate models, *D. melanogaster* and *C. elegans*, widely used in research for their genetic tractability, rapid life cycles, and suitability for large-scale modifier screens [12]. *D. melanogaster* is currently used to mirror ALS-associated symptoms by evaluating climbing and crawling, eye morphologies, and brain histologic profiles [13]. Likewise, *C. elegans* is widely used to analyze survival rate, the integrity of neuromuscular junctions, and cellular pathologies upon constructing a model with mutations in C9ORF72, SOD1, and TDP43 genes [14]. Unfortunately, both models raise concerns. In general, a major disadvantage of invertebrate models is that their use is fundamentally limited by evolutionary and anatomical differences from humans. The fly nervous system lacks the complex, layered cerebral cortex and upper/lower motor neuron circuitry characteristics of humans, making it impossible to directly study complex motor decline as it is present in patients. The metabolic system is vastly different, resulting in poor predictability of human drug absorption, distribution, metabolism, and excretion (ADME), and, consequently, high-throughput pharmacological screens often fail to translate to mammalian models or clinical trials. In addition, the flies lack an adaptive immune system, preventing researchers from studying the critical inflammatory components of ALS. Similarly, the key limitations in using *C. elegans* for ALS research are related to the lack of complex organs, a defined cardiovascular system, and an advanced central nervous system, making it impossible to study complex brain-wide pathologies, blood–brain barrier dynamics, or systemic inflammation. Finally, in worms, phenotypes are typically limited to basic crawling or swimming defects, failing to capture the full, progressive severity of human ALS [15].

The optical transparency and high genetic homology to humans render zebrafish (*Danio rerio*) a powerful ALS model. The major advantages rely on the following: (i) embryos and larvae optical transparency allowing researchers to use fluorescent markers to track motor neuron development and degeneration in real time without invasive procedures; (ii) genetic tractability as they share a highly conserved genome with humans, making them highly suitable for studying specific ALS genes (e.g., SOD1, TDP-43, C9orf72); (iii) high-throughput drug screening because they are small and develop quickly and hundreds of therapeutic compounds can be evaluated simultaneously in multi-well plates; (iv) cost and fecundity as they are significantly cheaper to house than rodents, mature in about 2 to 3 months, and produce hundreds of offspring per breeding cycle. However, there are notable limitations: as aquatic organisms, they lack the identical anatomical, physiological, and pharmacokinetic profiles of mammals, and differences in drug metabolism and disease progression exist; drug administration via the water medium makes exact dosage control difficult; and even if they exhibit complex motor phenotypes like swimming deficits, they do not possess the same higher-order cognitive or behavioural functions as mammalian models [16].

For neurodegenerative disease research, including ALS, mice are a key model as they closely mimic patient phenotypes, including bulbar and limb dysfunction. Because mouse and human anatomy are highly comparable, these models provide a strategic platform for preclinical drug testing and for exploring systemic tissue interactions, allowing researchers to study both local CNS networks (e.g., neuron–neuron and neuron–glia interactions) and peripheral pathways, such as the liver–brain axis, gut–brain axis, and immune-brain crosstalk, that are known to drive ALS progression [17]. The most widely used models are transgenic mice expressing SOD1 (G93A, G37R, G85R, and H46R), TARDBP, FUS, and C9orf72 mutations. Each of these displays advantages as well as disadvantages, as reported below.

One of the main advantages of SOD1-related ALS models is that mutations in the SOD1 gene were among the first genetic alterations identified in ALS and, so, results are supported by a substantial body of literature, and they are highly reproducible. Also, the introduction of different SOD1 mutations permits the generation of multiple transgenic animal models, allowing researchers to investigate the effects of distinct mutations on ALS pathogenesis while reproducing key pathological and clinical features of the disease, including motor neuron degeneration and progressive motor dysfunction. Despite these important strengths, SOD1-related models have several limitations: different SOD1 mutations can produce considerable phenotypic variability, making comparisons across studies and the interpretation of experimental findings more challenging; SOD1 mutations account for only a small proportion of all ALS cases and do not fully capture the broader pathogenic mechanisms underlying the majority of sporadic and non-SOD1-associated forms of the disease [17].

TDP-43 gene mutations and protein inclusions are widespread in ALS patients, making TDP-43 models highly relevant for capturing real-world disease diversity and pathology. However, their interpretation is complicated by the wide spectrum of TARDBP mutations, the structural heterogeneity of TDP-43 aggregates, and protein overexpression, which may produce non-physiological effects and experimental artefacts that do not accurately represent the molecular pathways driving neurodegeneration [17].

Mutations in the FUS gene are strongly associated with familial ALS and encompass a broad spectrum of genetic variants, rendering FUS-related ALS mouse models a valuable tool for investigating disease mechanisms. These models provide important insights into how disruptions of RNA metabolism, transcriptional regulation, and other aspects of RNA processing contribute to neurodegeneration. Nevertheless, the considerable phenotypic heterogeneity associated with different FUS mutations complicates the interpretation and comparison of experimental findings and makes it difficult to distinguish the primary pathogenic mechanisms from secondary downstream effects, thereby limiting the ability to attribute disease phenotypes to specific molecular alterations [17].

Finally, as one of the most frequent genetic causes of ALS, C9orf72 gene expansions give C9orf72 models exceptional clinical relevance due to the association with immune system activation, providing an excellent framework for studying neuroinflammation. Nevertheless, modelling C9orf72 pathology is complex, as the underlying mechanisms span multiple cellular domains, including RNA metabolism, immunity, and cytoplasmic mechanics. Additionally, similar to SOD1, C9orf72 mutations display high heterogeneity across patients, increasing the difficulty of translating research findings [17]. In recent years, transgenic large animal models, particularly swine, have emerged as an exciting paradigm to bridge the severe translational disconnect between rodent testing and human clinical trials, because their anatomy, brain structure, size, and genetic/immune profiles closely resemble humans compared to rodents. Swine models engineered to harbour ALS-associated mutations (such as SOD1 or TARDBP) offer substantial physiological and anatomical advantages over traditional rodent systems. Unlike lissencephalic mouse brains, the porcine brain possesses a complex gyrencephalic cortex, extensive white matter architecture, and a corticospinal tract that closely reflects the human motor neuraxis in scale, somatotopic organization, and neuronal somal dimensions. Crucially, their large CNS volume enables researchers to evaluate human-equivalent volumetric dosing and optimize delivery routes under realistic cerebrospinal fluid flow and pharmacokinetic dynamics. A pig model established by the overexpression of mSOD1 was reported for the first time in 2014, and the occurrence of motor neuropathy and neuronal degeneration has been clearly demonstrated [18,19]. Recently, Kondo et al. generated a pig model of human mutant SOD1-mediated fALS by using intracytoplasmic sperm injection-mediated gene transfer to deliver a genetic construct—containing the entire human mutant SOD1 locus alongside its promoter region—into porcine zygotes. This model exhibited early motor neuron abnormalities accompanied by the accumulation of misfolded SOD1 in the spinal cord prior to symptom onset. Furthermore, it demonstrated that misfolded SOD1 accumulates before motor neurodegeneration takes place, triggering abnormal glial responses and exerting strong neuronal cytotoxicity [20]. Finally, a porcine model of ALS via ear vein injection of an AAV-TDP-43M337V vector recapitulates key hallmark features of ALS, including pronounced motor impairment and behavioural deficits, accompanied by severe muscle atrophy and fibrosis. These pigs exhibit progressive spinal motor neuron degeneration driven by TDP-43 pathology, along with robust glial cell activation across the brain and spinal cord. Neurodegeneration depends on elevated levels of TMEM106B, SOD1, and APOE4 proteins alongside impaired lysosomal and autophagic pathways [21].

Despite these translational strengths, several major limitations constrain the widespread implementation of swine models in ALS drug discovery. Primary obstacles include exorbitant housing, dietary, and veterinary maintenance costs; extended gestation cycles and slow maturation rates that drastically lengthen experimental timelines; complex ethical and regulatory oversight; and a relative scarcity of validated, swine-specific antibodies, molecular reagents, and standardized behavioural testing batteries compared to the vast rodent infrastructure.

In addition to technical limits, ethical concerns about animal experiments for medical research are also present, as regulated by the 3Rs (Replacement, Reduction, and Refinement) principle, defined for the first time in 1959 and now actively integrated into European and global scientific policy. This principle requires scientists to do the following: substitute animals with non-animal alternatives like in vitro or computer models (Replacement); minimize the number of animals used to obtain statistically reliable data (Reduction); and minimize pain, stress, and suffering by improving animal housing and care (Refinement) [22,23]. Since 2022, in vitro evidence is sufficient for translating results to clinical trials; in addition, as of April 2025, the National Institutes of Health (NIH) do not fund grant proposals using exclusively animal models.

## 4. Cell Culture 2-D Models for ALS Research: Potential and Limits of Their Translatability

Cells grown in a dispersed monolayer (two-dimensional (2D) cell culture systems) are the simplest and most cost-effective tool for studying cellular mechanisms of diseases and have led to significant advances in understanding pathobiological mechanisms of neurodegeneration. Unfortunately, this method has limits to its translatability in complex diseases like ALS, in which multiple regions of the nervous system are affected; so, it is possible that 2D monocultures may provide false positives or false negatives when used to screen novel therapeutic agents.

The most employed cell culture systems to study ALS pathogenesis include immortalized cell lines, such as SH-SY5Y, NSC-34, or HEK293 cells, patient-derived fibroblasts and lymphoblasts, and primary neural and glial cells obtained from post-mortem tissue. The first two represent accessible and versatile platforms for their ease of culture and genetic manipulation, and for obtaining information about the localization of mutated proteins (SOD1, TDP-43, FUS), the oxidative stress pathways, autophagy, or RNA metabolism [24,25]. Primary cells provide interesting data on the vulnerability of neurons and their interaction with glial cells [26]. Unfortunately, these human-derived systems have specific limitations, including cellular diversity, physiological relevance, scarcity of materials, and impossibility of long-term maintenance. All of them restrict their translational potential [27].

Recent advances in biotechnology, mainly through the development of induced pluripotent stem cells (iPSCs) and CRISPR-Cas9 gene-editing technologies, have revolutionized the approach toward understanding and treating ALS. In particular, iPSCs have emerged as a key platform, effectively bridging the gap between simplified in vitro systems and the complex, multifactorial pathophysiology observed in ALS patients. iPSCs can be obtained from patient-derived fibroblasts or peripheral blood mononuclear cells (PBMCs) by the exogenous expression of four transcription factors (Oct3/4, Sox2, Klf-4, and c-Myc) that reprogram them back into an uncommitted stem cell phenotype [28]. Once generated, iPSCs can be differentiated into neural lineages, giving rise to multipotent neural stem cells as well as diverse mature neuronal subtypes, including MNs and cortical neurons, and glial cells, such as astrocytes, microglia, and oligodendrocytes, providing a unique model system to study disease mechanisms and test therapeutic interventions, as they have the advantage of maintaining the patient’s genetic background, thereby advancing personalized medicine approaches [29].

An important advantage of iPSC neurons is the possibility to make 2D co-cultures with other differentiated cell types, including glial cells and muscle cells, or to generate 3D organoids, thus mimicking the complexity of the human brain and the neuronal circuitry, as subsequently reported in Section 5.1.

While iPSC neurons are powerful tools, several critical limitations still hinder their widespread application, including the cost of maintaining cell cultures, the lack of unified protocols for both generation and differentiation, reprogramming efficiencies that vary widely, and the difficulty in replicating cellular ageing and long-term environmental exposures.

Taking into account that the CNS is very complex in terms of structure and function and the nervous tissue consists mainly of neurons and glial cells combined in a highly complex manner to form a three-dimensional (3D) architecture, a single-cell monolayer results in physiological or metabolic properties completely different from those in vivo. In addition, the multisystem nature of ALS cannot be studied with a single cell type, as cell-to-cell communication is pivotal to understanding motor neuron cell survival or degeneration, and the urgent need to study the interactions between multiple cell types, e.g., glial, peripheral immune system, muscle, and gut microbiota cells, in ALS is now widely accepted [9].

To overcome the drawbacks of single-cell cultures, cell co-culture systems can be useful, allowing the study of cell-to-cell communication and the interaction between cells and their surrounding microenvironment in terms of cell differentiation, viability, proliferation, and migration, both in direct and indirect contact. In this last case, different cell types can be plated in two different chambers of transwell systems characterized by a porous membrane, coated or not with molecules mimicking the extracellular matrix (ECM), permitting interaction through soluble factors present in the medium, called conditioned medium.

The major limitation of cell co-culture systems is represented by the fact that, in vivo, the cells are organized in a 3D structure that influences many cellular processes, like differentiation, gene expression, cell death, growth pattern, morphology, and function. For this purpose, advanced 3D models have been developed [30].

Table 1 summarizes the 2D human-derived cell culture models, their setup, major applications in ALS research, and the distinct advantages and limitations described in the text.

## 5. Next-Generation Models for ALS Research

An urgent need for realistic human model systems for recapitulating the brain tissue organization and function in ALS research exists to bridge the translational gap between preclinical study results and clinical application due to the limitations of 2D cell systems [31].

In general, cells and tissues cultured in a 3D environment show great potential to advance the understanding of complex biological processes. 3D cell models are increasingly being used in many research applications, including drug discovery, toxicology, disease modelling, and regenerative medicine. These models offer an opportunity to better understand complex biology in a physiologically relevant context, providing an in vitro representation of a tissue or organ.

Currently, organoids, chimeroids, assembloids, and microfluidics-based organ-on-chip platforms are reported as the next-generation models for neurodegenerative diseases, including ALS; each of them displays advantages and challenges as detailed below.

### 5.1. Organoids

Neural organoids better model ALS pathogenesis as they reflect the human brain’s complexity. In 2013, Lancaster et al. developed the first human brain organoids using 3D cell culture techniques, successfully generating complex models that incorporated retinal, choroid plexus, midbrain, and forebrain cell lineages [32]. Neural organoids constructed using human iPSCs combine human genetic backgrounds and 3D tissue architecture, providing the possibility to have multicellular complexity characteristic of the brain in a simple, manipulable manner [33]. This offers a valid tool for bench-to-bedside translation for clinical applications [34]. In general, the first step in the generation of an organoid is the reprogramming of somatic cells into iPSCs. In fibroblasts or peripheral blood mononuclear cells, OCT4, SOX2, KLF4, and cMYC genes are introduced [28]. These transcription factors permit the self-renewal capacity and pluripotency of iPSCs that can differentiate into any cell type characterized by the same genetic background as the donor, and this is very important for modelling genetic forms of ALS [35].

Two strategies, guided or unguided approaches, exist to generate neural organoids. The first generates specifically regionalized neural organoids, in which iPCS differentiate into specific regions of the nervous system using specific growth factors and signalling molecules to direct cell fate. Conversely, the unguided approach generates organoids relying on spontaneous self-organization of cells without added morphogens [36,37].

The guided approach uses precise morphogens and relative inhibitors (such as SMAD inhibitors, WNT, SHH modulators, BMP, TGF-β) or signalling molecules (EGF, neurotrophin 3, and brain-derived neurotrophic factor) at precise developmental time points to mimic embryonic tissue gradients able to direct cells toward a specific, predefined regional or organ-specific identity (e.g., cortical, retinal, or hypothalamic organoids). If the neural organoids obtained with the guided approach display high reproducibility, uniform structure, and robust generation of target cell types, they lack the broader multisystem complexity found in unguided systems. This limitation is overcome through separate guided organoids later fused into assembloids, as described below [38].

The unguided approach starts with iPSC aggregation into embryonic bodies (EBs), embedded in an extracellular matrix (like Matrigel) and grown in neutral media without external patterning cues, letting intrinsic self-organization drive morphogenesis. During aggregation into EBs, the presence of ROCK inhibitors is essential for cell survival; in approximately 5–7 days, cells spontaneously suppress differentiation towards mesodermal and endodermal lineages and activate the neuroectodermal one. After that, the differentiated cells are embedded for 7–10 days in Matrigel, and so the tissue nervous architecture appears. The nutrient distribution and oxygenation are ensured by placing the organoid culture on spinner flask bioreactors or orbital shaking plates, permitting progressive neuronal differentiation and migration until complete formation of cortical layers [39]. This approach presents pros and cons: it captures complex, broad cellular diversity and spontaneous multi-region interactions, but it suffers from high batch-to-batch variability and structural unpredictability between individual organoids [38].

Recent advances have refined organoid generation protocols to boost their quality and physiological relevance. Organoid size and survival are improved by integrating mesodermal progenitors that yield vascularized organoids with endothelial cells and pericytes forming primitive capillaries that ameliorate oxygen and nutrient diffusion [40]. In addition, with microfluidic organoid-on-a-chip platforms, it is possible to obtain better microenvironmental control, enabling automated morphogen delivery, real-time growth monitoring, and controlled drug screening [41].

Finally, to improve the validity of brain organoids as models for neuroinflammation and neurodegeneration, strategies for incorporating microglia cells into neural organoids have been developed, as microglia do not spontaneously appear in standard brain organoid cultures due to different germ layer derivation of microglia and neurons. To achieve this, researchers can introduce iPSC-derived microglia-like cells into existing organoids, co-culture hematopoietic and neural progenitors from day one, or transplant primary human microglia into mature organoids [42,43].

In ALS research, patient-derived iPSCs are better to use in organoid generation as (i) they are more effective in expressing ALS phenotypes and (ii) they can overcome the restricted access to presymptomatic patient samples.

The main organoid types used in ALS studies are neuromuscular organoids, spinal cord organoids, and cerebral or cortical organoids. They differ primarily in their cellular makeup, anatomical region modelled, and functional connectivity. Neuromuscular organoids combine spinal motor neurons, skeletal muscle tissues, and supporting glia to form functional NMJs, and they are used to study contraction weakness, neural denervation, and synaptic instability. Spinal cord organoids focus on anterior horn motor neurons and central neuroglia and help researchers investigate cell-autonomous motor neuron degeneration and nuclear TDP-43 mislocalization. Finally, cerebral or cortical organoids mimic the human cerebral cortex and upper motor/cortical neuron networks and are utilized to track early molecular pathologies, dipeptide repeat protein (DPR) aggregation from C9ORF72 expansions, and cortical circuit changes [44].

#### 5.1.1. Cerebral or Cortical Organoids

Today, cortical organoids have become key tools for modelling ALS due to the air–liquid interface culture technique that, by optimizing oxygen delivery, permits organoids to achieve extended neuronal survival (exceeding 12 months), foster axonal outgrowth, and establish active neural networks [45]. Szebényi et al. established a cortical organoid model using ALS patient-derived iPSCs carrying the C9orf72 hexanucleotide repeat expansion, providing a human-specific platform to map the precise timeline and causative relationships underlying synaptic breakdown and neuronal loss in ALS [43]. Cultured for up to 240 days, these organoids developed a consistent microarchitecture with mature circuit-forming cortical neurons, and, even in the absence of microglia and vasculature, they successfully recapitulated early cell-type-specific pathology, including neuronal integrity and pathological markers, astrocytic involvement, and therapeutic targets. In particular, deep-layer neurons accumulate toxic poly-GA dipeptide repeat (DPR) proteins, exhibit elevated DNA damage, and undergo cell death. Single-cell RNA sequencing and functional assays further reveal disruptions in transcriptional regulation, proteostasis, and DNA repair mechanisms in neurons and astroglia. In particular, astrocytes display significant accumulation of p62, a marker associated with impaired autophagy signalling. Finally, pharmacological intervention targeting proteostasis helps mitigate neurodegeneration. Specifically, treatment with GSK2606414, a selective inhibitor of the unfolded protein response (UPR) mediator PERK (protein kinase R-like endoplasmic reticulum kinase), inhibits cell death in the model [43].

#### 5.1.2. Spinal Cord Organoids

iPSC-derived spinal cord organoids provide a powerful platform to study lower MN pathology in ALS, as by combining 3D culture alongside high-throughput sequencing techniques, such as bulk and single-cell RNA sequencing (scRNA-seq), researchers can track disease progression in vitro and explore cell-type-specific pathology involving both neuronal and non-neuronal cells [46]. To investigate the role of C9orf72 deficiency in ALS, Guo et al. generated a loss-of-function model by stably knocking down C9orf72 expression in healthy wild-type (WT) iPSCs that retained pluripotency and efficiently differentiated into astrocytes and MNs. Spinal cord patterning was achieved through activation of Wnt signalling, inhibition of bone morphogenetic protein (BMP) signalling, caudalization with retinoic acid, and ventralization using purmorphamine, a Sonic Hedgehog (Shh) agonist. This protocol produced C9-knockdown spinal cord organoids that effectively mirrored native spinal cord neurogenesis and were able to form a neural tube-like structure characterized by a central canal and diverse cell populations, including motor, sensory, and interneurons. Transcriptomic analyses demonstrated significantly increased expression of pro-inflammatory cytokines, including IL6 and TNFα, in organoids compared to conventional 2D cultures of motor neurons or astrocytes. Bioinformatic analyses further revealed that although inflammatory pathways were activated in both culture systems, the 3D organoid environment markedly amplified the neuroinflammatory response, highlighting the importance of multicellular interactions in the pathogenesis of C9-ALS [46].

#### 5.1.3. Neuromuscular Organoids

Skeletal muscle dysfunction is considered an important mechanism in the pathogenesis of ALS, as demonstrated by two prominent and competing theories regarding disease origin and spreading, the “dying-forward” and the “dying-back” hypotheses, which attempt to explain the involvement of the neuromuscular junction (NMJ) in the degeneration of both upper motor neurons (in the brain) and lower motor neurons (in the spinal cord). The first hypothesis, “dying-forward”, posits that ALS starts in the brain, specifically within cortical motor neurons that become hyperactive, leading to a glutamate-driven anterograde (forward) excitotoxic process. This continuous overstimulation damages the connections between upper and lower MNs and causes the dysfunction and death of spinal motor neurons, and so the NMJ dismantling. The “dying-back” hypothesis states that ALS is a distal axonopathy: degeneration begins at the NMJ or within the muscle itself, and a retrograde signalling cascade leads to motor neuron death [47].

To investigate these early peripheral mechanisms, iPSC-derived neuromuscular organoids have recently been developed to model the spinal neuromuscular system in ALS. Neuromuscular organoids generated from patients carrying the C9orf72 hexanucleotide repeat expansion (HRE) faithfully reproduced several peripheral hallmarks of the disease, i.e., impaired muscle contractility, motor axon denervation, and Schwann cell loss [48]. Disease-associated RNA foci and dipeptide repeat proteins (DPRs) were detected in both neurons and astrocytes, with astrocytes exhibiting more pronounced DPR aggregation. This cellular distribution differs from that reported in C9orf72 brain organoids, where poly-GA aggregates were largely confined to cortical neurons, highlighting potential tissue-specific differences in disease pathology that warrant further investigation. Importantly, the translational utility of neuromuscular organoids was demonstrated by treatment with the unfolded protein response (UPR) inhibitor GSK2606414, which approximately doubled muscle contractile force while reducing autophagy and DPR accumulation [48]. Bakooshli et al. generated functional 3D NMJs by combining human skeletal muscle progenitors with pluripotent stem cell-derived MNs, although this model did not incorporate ALS patient-derived cells [49]. Continued refinement of these platforms will be critical for faithfully reproducing physiological synapses and exploiting them for mechanistic studies and preclinical drug discovery. More recently, advanced sensorimotor organoids containing physiologically functional NMJs have expanded the complexity of ALS disease models [50]. Generated from iPSC lines derived from healthy individuals and patients with ALS, these organoids were cultured for up to 15 weeks and developed integrated neuromuscular tissues containing skeletal muscle, MNs, astrocytes, microglia, vascular-like cells, and functional NMJs. Electrophysiological, molecular, and immunocytochemical analyses demonstrated that organoids carrying ALS-associated mutations exhibited early NMJ abnormalities, reinforcing the concept that synaptic dysfunction represents one of the earliest pathogenic events in ALS. The generation of isogenic control lines harbouring mutations in TARDBP, SOD1, and PFN1 substantially reduced experimental variability and enabled more precise dissection of mutation-specific disease mechanisms, thereby strengthening the utility of this platform for mechanistic investigations and therapeutic screening [50].

### 5.2. Chimeroids

Chimeroids represent a novel class of multi-donor human brain organoids engineered to model inter-individual genetic heterogeneity within a unified three-dimensional tissue microenvironment [51]. Chimeroids eliminate culture-to-culture batch variability and provide a robust humanized system for high-content screening, candidate drug validation, and personalized therapeutic targeting in ALS. In fact, rather than aggregating undifferentiated human iPSCs, which typically leads to clonal dominance by fast-proliferating lines, chimeroids are generated by growing individual donor iPSC lines into single-donor neural progenitor cells before dissociation and re-aggregation into chimeric neural assemblies, allowing multiple genetic backgrounds [51]. In the context of ALS, chimeroids could offer a powerful high-throughput platform to dissect patient-specific vulnerability, as by co-culturing cell lineages derived from healthy donors alongside patients carrying distinct familial mutations (e.g., C9orf72, SOD1, TARDBP) or sporadic ALS phenotypes within the same organoid, researchers could directly compare pathological hallmarks, such as TDP-43 mislocalization, axonal degeneration, and neuroinflammatory astrocyte activation, under the same microenvironmental conditions [51].

### 5.3. Assembloids

The generation of organoids represents significant progress in overcoming the challenges associated with directly studying the human central nervous system within a physiologically relevant microenvironment. However, since cell fate specification in regionalized neural organoids primarily relies on the addition of specific signalling molecules to the culture medium (guided differentiation), these models tend not to produce multiple niches spontaneously. Consequently, individual organoids often fail to consider interactions between distant brain regions or communication across different germ layer lineages. To address these limitations, research is evolving toward the creation of assembloids, structures formed by fusing multiple region-specific organoids, allowing for the modelling of long-range axonal projections and complex network dynamics. Assembloids can be grouped into two main categories: multi-region and multi-lineage. Multi-lineage assembloids form when organoids merge with specialized cell types derived from differentiated stem cells in 2D cultures or primary tissue-derived stem cells [52,53]. Birey and colleagues introduced forebrain assembloids to model how ventrally derived interneurons migrate into dorsal cortical areas, yielding new insights into early human brain development [54]. Based on that, Andersen and colleagues in 2020 generated the first 3D cortico-motor assembloid by associating cortical, spinal, and muscle spheroids [55]. The generated 3D model shows cortical MN projections towards spinal spheroids whose MNs connect, in turn, with muscle. Andersen demonstrated that the circuit is physiologically functional up to 10 weeks post-fusion, as stimulation of cortical spheroids causes contraction of muscle [55]. These models offer a strong platform for examining motor and neuromuscular disorders and the complex interactions between neurons and muscles.

In the context of ALS research, assembloids are particularly instrumental for modelling the “motor neuraxis,” recreating the human pyramidal tract effectively by integrating cortical, spinal cord, and skeletal muscle tissues. These cortico-motor assembloids allow for investigating ALS-specific hallmarks that cannot be captured in simpler models, including impaired corticospinal connectivity, synaptic hyperexcitability, and NMJ denervation. Moreover, it is possible to construct assembloids able to mimic the gut–brain axis in which complex interactions between the nervous system, endocrine system, immune system, and gut microbiota exist.

In various neuroinflammatory conditions, a pivotal role is played by immune cells, including peripheral macrophages and resident microglial cells [56,57,58,59].

Current organoid protocols still lack the microglial component; in turn, even the assembloids do not have it. Researchers are trying to develop new protocols to generate assembloids that can mimic nervous tissue in all its components. A study by Kalpana in 2024 proposed two alternative approaches to generate neuroimmune assembloids using iPSC-derived cortical organoids and microglia. The first approach involves direct incorporation of microglia into the organoids; the second suggests combining microglia and neural progenitors in a specific ratio before the generation of the organoid [60]. By incorporating patient-derived glial cells—such as astrocytes or microglia harbouring specific mutations in C9orf72, SOD1, or FUS—assembloids enable the precise dissection of non-cell-autonomous toxicity and neuroinflammatory propagation. Furthermore, these models allow researchers to evaluate cell type-specific metabolic dysfunction and identify prospective therapeutic targets within a humanized, highly relevant microenvironment.

### 5.4. Microfluidics-Based Organ-on-Chip Platforms

While protocols exist for generating organoids of various systems, integrating the neural system with other tissues remains challenging. This difficulty stems from the dynamic and intricate physiological microenvironment connecting different organs. A promising solution is the organ-on-a-chip—a microfluidic platform featuring dedicated compartments for distinct organ models [61,62].

This capability is essential for ALS, a disease that propagates through a specific circuit—the corticospinal tract—spanning the brain, spinal cord, and periphery, and involving distinct cell types (upper/lower motor neurons and skeletal muscle). So, the platforms allow for replicating neuromuscular junctions, revealing how axons degenerate, and testing drug treatments in human-relevant microenvironments by exploiting iPSC technology [63]. On these devices, it is possible to grow iPSCs from patients with SOD1, FUS, or TDP-43 mutations (differentiated directly on chip into MNs, muscle cells, etc.) in a microfluidic system. In this manner, different cell types involved in ALS pathophysiology are separated into chambers connected by microchannels, in which fluid mimics blood flow, permitting to investigate how the communication nerve-muscle fail over time, reveal abnormal glutamate activity in ALS cells and understand how the disease kills nerves, test candidate medicines on human diseased tissue before human trials [64].

Micro-etched channels (typically 500 nm–1 µm in diameter) characterize microfluidic devices containing microvolumes of fluid. A primary advantage of microfluidic modelling lies in its architectural flexibility, allowing systems to be customized for specific research objectives. The chip can incorporate dedicated cellular compartments connected by microchannels for studying interactions between spatially separated distinct cell populations sharing the same microenvironment [65,66]. Additionally, analytical components such as multielectrode arrays (MEAs) can be integrated directly into the fabrication process to enable real-time electrophysiological recording [67]. Polydimethylsiloxane (PDMS) remains the gold standard for microfluidic fabrication due to its optical clarity, hydrophobicity, gas permeability, affordability, and ease of sterilization. Alternatively, devices can be fabricated from thermoplastics, silicon, or cellulose-based papers [68]. To minimize device-to-device variability, microfluidics are commonly produced via mould casting—a process where liquid PDMS is poured onto a master mould, cured, peeled off, and bonded to a glass coverslip to form enclosed fluidic pathways [69]. In neurobiological applications, devices typically feature three core elements: inlet and outlet wells for media delivery, culture chambers for cell residence, and interconnecting microchannels. Suspended cells introduced at the inlet wells are drawn into the chambers via capillary action. A critical capability of these platforms is the generation of controlled fluid flow and concentration gradients via external pumps or through hydrostatic pressure and capillary forces established by volume differentials [70]. This continuous perfusion provides sustained nutrient delivery and waste clearance, closely mimicking the physiological circulation of cerebrospinal fluid (CSF) in vivo.

In ALS research, CSF dynamics are critical both for understanding how pathological drivers disseminate through the CNS and for designing targeted drug-delivery strategies. Microfluidic architectures excel at establishing precise chemical gradients by introducing solutes, growth factors, or therapeutics at localized region boundaries, allowing stable diffusion profiles to form passively. For example, Chennampally et al. applied a rapamycin gradient across iPSC-derived motor neurons harbouring the ALS-associated TARDBP A315T mutation. This approach delineated a clear therapeutic window: rapamycin concentrations between 0.4 and 1 µM rescued 40.44% of motor neurons, whereas lower doses proved ineffective and higher doses proved cytotoxic [63]. Notably, low-dose rapamycin has been demonstrated to be safe in a phase 2 clinical trial for ALS patients [71]. While microfluidic platforms offer valuable versatility for mechanistic ALS research, their current translation to high-throughput drug screening faces notable challenges, including lack of standardization, material limitations, physiological stressors, and operational complexity. Widespread variation exists across device geometries, fabrication workflows, and cell-handling protocols (including surface functionalization, seeding density, co-culture media composition, and flow control). High-throughput screening requires standardized, highly reproducible models over bespoke designs. PDMS readily sequesters hydrophobic small molecules, and although mitigation strategies exist, they remain underutilized in screening workflows [72]. Furthermore, compartmentalization introduces physical challenges rare in standard 2D cultures, including fluid shear stress, altered gas exchange dynamics, and modified nutrient depletion kinetics [73]. Finally, translating traditional 2D culture assays to microfluidic systems requires re-optimizing seeding protocols, feeding schedules, and media formulations. Without widespread automation in academic settings, these manual workflows remain labour-intensive and require specialized expertise.

In ALS, the study of NMJ dismantling is particularly interesting to counteract the clinical signs of disease. In fact, while motor neurons are the primary targets, symptom onset is fundamentally driven by the degradation of NMJs. Osaki et al. engineered a human-derived microfluidic model to recapitulate this complex interaction in vitro, offering a scalable, highly accurate platform for discovering much-needed therapeutics. Human iPSC-derived skeletal muscle cells were seeded in a microfluidic chamber around flexible pillars, forming 3D muscle fibre bundles, and contraction forces were quantified by measuring pillar deflection, while human ESC-derived motor neurons and astrocytes were co-cultured in an adjacent chamber, and MNs were optogenetically engineered via channelrhodopsin-2 (ChR2) expression to enable light-driven action potentials. Axons extended across a collagen barrier into the muscle chamber, establishing functional NMJs within 4 days. By day 14, thickened nerve fibres induced synchronized muscle contractions upon optogenetic or neurotransmitter stimulation. Using iPSC-derived MNs from a patient with sporadic ALS harbouring a pathogenic TARDBP/TDP-43 mutation and cytoplasmic TDP-43 aggregates, Osaki’s team observed significant NMJ pathology relative to healthy controls. In particular, lower axon outgrowth, reduced formation of thick nerve fibres, and a drop in responsiveness from ~100% to ~60% were observed. In the same way, muscle contraction force plummeted to ~25% of control levels by day 14, and elevated apoptotic markers indicated progressive muscle fiber death [74]. Taking into account these results, the researchers investigated the feasibility of the chip also for studying a potential therapeutic approach. Treating the diseased chip with rapamycin (an autophagy inducer) combined with bosutinib (a Src/c-Abl inhibitor) cleared toxic TDP-43 aggregates from the cytoplasm. This combination therapy outperformed single-drug treatments, dramatically restoring neuronal firing, rebuilding muscle contraction force, and preventing cell death [74].

Turco and coworkers constructed a lab-on-chip electrochemical biosensor for rapid, point-of-care quantification of TDP-43. The platform leverages electrosynthesized polypyrrole derivatives bearing carboxyl functional groups for site-specific anti-TDP-43 antibody immobilization. The biosensor demonstrated rapid response times, strong reproducibility, and a broad linear dynamic range with a limit of detection of 10 pg/mL. The chip successfully quantified TDP-43 levels in human serum samples from both ALS patients and healthy controls, demonstrating that it is a robust, cost-effective diagnostic tool with strong potential for early ALS clinical screening and therapeutic drug monitoring [75].

Finally, a microfluidic spinal cord chip that co-cultures early-onset ALS patient-derived MNs with brain microvascular endothelial-like cells under physiological fluid flow has been designed by Lall in 2025, demonstrating that media flow significantly enhanced neuronal maturation and cellular viability. Integrated bulk transcriptomic and proteomic profiling identified distinct molecular signatures differentiating ALS from control lines; single-nucleus RNA sequencing resolved two distinct MN subpopulations and revealed ALS-specific dysregulation of glutamatergic transmission and synaptic signalling. By combining a functional, blood–brain barrier (BBB)-like microvasculature with a diverse population of mature MNs, this chip provides an advanced human platform for elucidating ALS pathophysiology and accelerating high-fidelity drug screening [76].

In Table 2, a summary of key characteristics, advantages, limitations, and potential solutions of the next-generation in vitro 3D cell models is reported.

## 6. CRISPR-Cas9 Technology as New Hope in ALS Modelling and Therapies

The complexity of ALS—where only 10% of cases are inherited (fALS)—presents a significant challenge; however, several biotechnology firms are advancing CRISPR-based solutions, and they are now beginning to be used in clinical settings. For the majority of patients with sporadic ALS (sALS), technologies like CRISPR inhibition (CRISPRi) may be used to “switch off” problematic proteins that contribute to the disease even in the absence of a clear genetic mutation.

CRISPR/Cas9 (Clustered Regularly Interspaced Short Palindromic Repeats- associated protein 9) is a technology developed based on an adaptive immune mechanism found in bacteria, where it functions to detect and destroy invading bacteriophage DNA following an infection. Much like a molecular cut-and-paste tool, the system enables targeted genome editing. While alternative technologies exist—such as Zinc Finger Nucleases (ZFNs) and Transcription Activator-Like Effector Nucleases (TALENs)—CRISPR/Cas9 is widely preferred due to its simplicity, efficiency, and cost-effectiveness [77]; its gene-editing ability was confirmed in human cells in 2013 [78]. In bacteria, upon infection, fragments of the invading DNA are integrated into the host’s CRISPR array to create a genomic memory; the resulting transcripts will serve as a molecular guide if the organism encounters the same foreign genetic material again and direct the Cas endonuclease to precisely recognize and cleave the matching invader [79]. Although very promising, potential safety concerns exist, and several strategies to overcome this have been developed [80,81]. Currently, for genetic disorders like ALS, CRISPR/Cas9 is widely deployed to generate cell and animal models and develop therapies, also by combining it with iPSC technology. This dual approach helps validate the pathogenic role of specific variants while uncovering novel targets for therapeutic intervention. By correcting underlying disease-causing variants, gene-editing technologies hold strong therapeutic potential for ALS and are actively transitioning into clinical applications [82].

Recent advances in CRISPR/Cas9 technology enable researchers to introduce precise genomic modifications across diverse cell lines, including HT22, BV2, Neuro-2a, NSC-34, hESC, and HeLa lines, allowing the generation of targeted cell models for investigating distinct ALS pathologic mechanisms. For instance, Liu et al. (2022) utilized CRISPR/Cas9 to generate an astrocyte elevated gene-1 (AEG-1) deficient HT22 cell model, providing a robust platform to investigate AEG-1-mediated ALS pathology [83]. Zhang and coworkers) employed CRISPR/Cas9 to introduce the UBQLN2 P497H mutation into a healthy control iPSC line and, upon differentiation into motor neurons, they created a targeted model to elucidate the pathological mechanisms underlying UBQLN2-associated ALS [84]. Further, Strohm engineered HeLa cells carrying UBQLN2 mutations to evaluate how these variants drive neurodegeneration [85]; Prtenjaca, focusing on OPTN mutations, typically triggering cytoplasmic mislocalization, ubiquitination, and accumulation of nuclear TDP-43, generated OPTN knockout lines in Neuro-2a, NSC-34, and BV-2 cells, demonstrating that optineurin depletion leads to elevated TDP-43 levels specifically in microglia [86]. Similarly, a heterozygous FUS-Q290X knock-in human embryonic stem cell line has been designed by Zhang [87].

In addition to ALS cell models, CRISPR/Cas9 is helpful in generating animal models, both invertebrates, such as *Drosophila melanogaster*, *Danio rerio*, *C. elegans*, and vertebrates, including mice, as summarized in Table 3.

The majority of causative genetic mutations in ALS, both familial and sporadic forms, are related to SOD1, C9orf72, FUS, and TARDBP genes. CRISPR/Cas9 technology is currently being exploited to correct the mutant DNA to eliminate abnormal downstream pathways and identify potential therapeutic targets. Unfortunately, few studies are still available.

Several studies highlight the utility of CRISPR/Cas9 technology in both patient-derived iPSCs and mouse models in mitigating SOD1 mutation-induced toxicity.

Wang et al. (2017) established two iPSC lines from fibroblasts of familial ALS patients carrying disease-causing mutations SOD1^+/A272C^ and SOD1^+/C14T^, and successfully generated corresponding isogenic controls using CRISPR/Cas9 gene correction, revealing 899 aberrant transcripts and providing a promising platform for early biomarker discovery and therapeutic development in ALS [101]. The team of Imamura utilized CRISPR/Cas9 to correct the SOD1^L144FVX^ mutation in iPSC lines derived from an ALS patient. The gene-corrected cells reverse pathological ALS phenotypes, restoring motor neuron survival and intracellular ATP levels, and promoting the clearance of misfolded SOD1 protein. Furthermore, the study demonstrated that bosutinib, a Src/c-Abl inhibitor, prolongs survival in a SOD1-mutant ALS mouse model, highlighting the Src/c-Abl pathway as a promising therapeutic target for ALS [102]. Finally, isogenic iPSC lines obtained by correcting the E100G mutation in the *SOD1* genomic locus using CRISPR-Cas9 genome editing technology have been used to uncover additional pathways dysregulated in ALS MNs. Pathway analysis and drug screening identified active ERK and JNK signalling as primary drivers of SOD1-mutant MN degeneration mediated by elevated expression of the downstream target JUN relative to non-MNs. Additionally, mutant FUS MNs showed activation of ERK and p38, highlighting a shared set of druggable pathways across distinct ALS mutations [103].

CRISPR/Cas9 technology allows for investigating the efficacy of correction of the SOD1 G93A mutation even in a mouse model. For example, Gaj demonstrated that genome editing knocked down mutant SOD1 protein levels by over 2.5-fold in the thoracic and lumbar spinal cord, preserved motor function, reduced muscle atrophy, and increased end-stage motor neuron survival by ~50%. Compared to controls, treated mice experienced a ~37% delay in disease onset and a ~25% lifespan extension [104]. Similarly, several studies demonstrate that by modifying mutant SOD1 in SOD1G93A transgenic mice, both the lifespan and performance of animals improve with restoration of muscle atrophy [105,106,107].

As reviewed by Shi in 2023, for C9orf72, FUS, and TARDBP mutations, multiple studies conducted on human iPSCs about the efficacy of CRISPR/Cas9 genome editing in reducing the expression of mutated genes and ameliorating neurodegeneration signs exist [108].

Companies like Scribe Therapeutics and Biogen, as well as Capsida Biotherapeutics and CRISPR Therapeutics, are developing in vivo therapies with a focus on improving delivery to increase safety and effectiveness. Finally, for the majority of patients with sporadic ALS, technologies like CRISPR inhibition (CRISPRi) may be used to “switch off” problematic proteins that contribute to the disease even in the absence of a clear genetic mutation.

## 7. How 3D Bioprinting Can Revolutionize ALS Research

3D bioprinting technology represents a rapidly evolving frontier that bridges conventional tissue engineering and advanced organoid modelling. 3D bioprinting technology utilizes bioinks, combinations of diverse cell types and biocompatible materials, dispensed via a bioplotter, to fabricate physiological tissue constructs. This approach supports cell viability within a native-like 3D microenvironment, enabling more accurate modelling of tissue-level biology, cell–cell interactions, and cell–matrix dynamics. Since Guillemot defined the field in 2010 [109], a variety of biomaterials have been developed for 3D constructs, including hyaluronic acid, xyloglucan, collagen, sodium alginate, gelatin, nanofibers, and carbon-based matrices [110]. While significant strides have been made in printing vascularized, osseous, chondral, and cardiac tissues, neural bioprinting remains emerging, and progress is largely limited to small structural applications, like nerve grafts, with disease modelling remaining difficult to obtain.

Furthermore, 3D bioprinting has emerged as a powerful platform to model central and peripheral nervous systems; in the context of ALS pathogenesis, it could be capable of recreating human-relevant multicellular spinal cord and neuromuscular environments. Unlike traditional 2D cultures, similar to organoids, bioprinted models capture cell–cell interactions, extracellular matrix cues, and tissue-level architecture that drive key ALS phenotypes such as MN degeneration, NMJ failure, and glial-mediated toxicity. Scarian in 2022 by integrating iPSC differentiation with 3D bioprinting establish a more physiologically relevant model for neurodegenerative diseases. Peripheral blood mononuclear cells from control subjects and sporadic ALS patients were reprogrammed into iPSCs and initially differentiated into neural stem cells in 2D culture before their encapsulation within a hydrogel-based bioink, 3D bioprinted, and sequentially differentiated into motor neuron progenitors and mature motor neurons within the 3D construct. The authors demonstrated that 3D-printed healthy and sALS lines maintain high cell viability throughout the 3D differentiation protocol, and the hydrogel matrix did not impair lineage-specific differentiation or cell electrophysiological functionality. These findings permit the authors to state that 3D bioprinting is a robust, physiologically relevant model for investigating ALS pathogenesis [111]. Stiffness, porosity, and biochemical gradients in bioprinted hydrogels better mimic the spinal cord ECM and influence MN survival, axon growth, and stress responses. 3D constructs can form synapses, NMJs, and even multi-synaptic corticospinal–muscle circuits, enabling readouts like contraction strength and electrophysiology.

While neural bioprinting was historically constrained to simple structural constructs like nerve grafts, recent developments in 2025 have driven the field toward printing vascularized, multicellular neural tissues. As highlighted by Choi et al., modern neural bioprinting leverages multi-head extrusion and stereolithography to co-print iPSC-derived motor neuron progenitors alongside supportive biofunctional matrices, fostering axonal guidance and cellular organization [112].

Crucially, integrating 3D bioprinting with organoid culture directly addresses major structural bottlenecks of unguided organoid systems, including core necrosis caused by restricted oxygen/nutrient diffusion and high batch-to-batch structural heterogeneity. The automated deposition of bioinks allows the creation of defined microchannels and bioengineered ECM constructs, facilitating continuous perfusion and highly reproducible differentiation of patient-derived motor neurons and glia for target identification and high-content drug screening. A team at Uppsala University used a 3D printer and ALS human stem cells generated from skin and programmed to become motor neuron progenitors, mixed with a soft gelatine, to create a model similar to human nerve tissue, making it possible to test new drug treatments in a lab environment. The three-dimensional distribution of the cells in the bioink material improved cell survival and growth of the nerve fibres [113].

The developed model is an optimization of a spinal cord organoid generated by the same team in which the neurites only grew on the surface of a gelatine-based scaffold. To solve this limitation, researchers use a softer 3D printing material (bioink) that retains its shape while still allowing outgrowth of the neurites in the material. Further, to sustain the cells’ maturation and differentiation, small mesoporous silica particles infused with growth factors and mixed into the bioink material were used [114].

These features make 3D bioprinted models especially valuable for studying sporadic ALS, where patient-specific genetic and environmental factors converge.

Despite rapid progress, key challenges remain: (i) as ALS is an adult-onset disease, accelerating or faithfully modelling ageing in bioprinted tissues is still difficult; (ii) creating stable, perfusable vasculature in thick neural constructs to support months-long cultures is an active area of development; (iii) fully incorporating microglia, peripheral immune cells, and meningeal/CSF-like compartments in a controlled way is technically demanding but crucial for neuroinflammation studies; (iv) variability in bioinks, printing parameters, and differentiation protocols can affect reproducibility across labs.

## 8. Can Omics Technologies and Machine Learning Models Help Diagnose ALS?

Identifying reliable biomarkers remains essential for ALS clinical practice. By combining multi-omics technologies with AI-driven machine learning, researchers can detect early disease patterns in complex biological data. Integrating diverse multi-omics and preclinical data through these advanced computational models clarifies the genetic and molecular mechanisms behind ALS pathogenicity. Ultimately, this approach streamlines drug discovery by identifying high-conviction therapeutic targets, significantly reducing costly failure rates in clinical trials.

Omics technologies, including genomics, transcriptomics, proteomics, and metabolomics, have transformed ALS research by shifting the focus from single-gene studies to system-level analyses. By capturing whole-genome sequences (genomics), gene expression profiles (transcriptomics), protein alterations (proteomics), and metabolic changes (metabolomics), multi-omics approaches decode the complex heterogeneity of ALS. For the first time in 2016, Caballero-Hernandez emphasized the urgent need to integrate clinical imaging with multi-omics data to enable early, accurate ALS diagnosis and guide rational drug design [115].

AI-driven computational models synthesize diverse multi-omics and (pre)clinical datasets to build a comprehensive map of ALS pathogenicity. By clarifying disease mechanisms, these models streamline drug discovery and identify high-conviction therapeutic targets, drastically reducing failure rates in subsequent clinical trials.

As an evolving discipline within applied mathematics, machine learning leverages algorithmic frameworks and training datasets to drive autonomous software learning. Its application to medicine, mainly in the oncology field, holds substantial promise for diagnostic precision, prognostic modelling, and patient risk stratification [116].

Machine learning could help researchers diagnose ALS earlier, identify hidden biological subtypes, and predict disease progression using complex genetic and imaging data. It analyzes blood samples for gene expression changes to detect ALS before traditional symptoms become clear; and coupled with MRI scans, it processes brain metrics to estimate ALS risk and severity in real time, cutting down the long waiting time required for an official neurological diagnosis. In this manner, recognizing specific patterns helps scientists design treatments tailored to a patient’s exact cellular profile rather than treating ALS as a single uniform disease; combining clinical variables, biofluid data, and genetic markers to forecast how fast the condition advances will permit proposing new ideas for clinical testing [117].

A breakthrough study led by the National Institutes of Health (NIH) demonstrated that multi-omics data combined with machine learning can detect ALS up to a decade before symptom onset. By screening nearly 3000 plasma proteins, researchers identified 33 proteins significantly altered in ALS patients, pointing to early pathological changes in skeletal muscle, nerve tissue, and energy metabolism. Among patients carrying the C9orf72 hexanucleotide expansion, eight specific proteins (including EIF2S2, HPCAL1, JPT2, MTIF3, PDAP1, and SMAD3) were markedly elevated, highlighting their potential as markers of early disease progression. To turn these molecular signatures into a clinical diagnostic tool, the team evaluated 10 machine learning algorithms and selected a Random Forest classifier incorporating 17 plasma proteins alongside demographic factors (age, sex) and sample collection conditions. The model achieved exceptional performance with an AUC of 96.2% and a balanced accuracy of 89.3% in discovery cohorts. When validated across a massive external cohort of over 23,000 individuals, the model maintained an average diagnostic accuracy of 98.3%, successfully differentiating ALS from other neuromuscular conditions. Crucially, the model generated an “ALS risk score” correlated with time-to-symptom-onset, revealing compensatory biological changes occurring up to 10 years presymptomatically [118].

Also, Michigan Medicine researchers developed an RNA sequencing-based machine learning model to enable early ALS diagnosis from routine blood samples. Rather than relying on a single biomarker, the team constructed a multi-gene classifier, a strategy adapted from oncology diagnostic panels. Analyzing differential gene expression revealed over 2500 dysregulated genes in ALS patients compared to healthy controls, with strong enrichment in immune pathways. Training an XGBoost 3.4.2 model on condensed gene panels (27–46 genes) yielded diagnostic accuracies up to 91%. Importantly, the analysis identified “core genes” in peripheral blood that mirror molecular alterations in spinal motor neurons. Leveraging these shared pathways, researchers prioritized eight potential therapeutic candidates, including the antipsychotic trifluoperazine and the BTK inhibitor ibrutinib, laying the groundwork for future clinical validation [119].

Finally, to map the genetic and molecular architecture of ALS, the team coordinated by Poelmans integrated multi-omics data using two complementary modelling frameworks: the Molecular Landscape Building approach and the Patrimony platform. Operating on distinct datasets, analytical methods, and scoring algorithms, this orthogonal strategy yielded strong convergence in both validating established therapeutic targets and identifying novel candidates. Of 217 targets currently or previously evaluated in clinical pipelines, 10 achieved top-tier scores across both models, confirming their high pathological relevance. Furthermore, the dual-model framework prioritized novel putative targets, notably highlighting MATR3, a protein with robust genetic, molecular, and functional ties to ALS pathogenesis [120].

## 9. Conclusions

The translation of preclinical findings into effective clinical therapies for amyotrophic lateral sclerosis (ALS) remains heavily hindered by the physiological limitations of standard non-human and traditional 2D cell culture models. Standard 2D cultures oversimplify cellular architecture, while animal models fail to recapitulate human-specific pathogenic mechanisms and genetic backgrounds.

Despite decades of preclinical drug discovery efforts, the clinical translation rate in ALS remains remarkably dismal, with over 90–95% of candidate therapeutics failing in Phase II/III human trials after demonstrating preclinical efficacy in standard transgenic rodent models (primarily SOD1-G93A mice). This catastrophic attrition rate stems from profound interspecies pharmacokinetic and metabolic disparities, the absence of TDP-43 pathology in SOD1 models, and the inability of simplified 2D cell monocultures to reflect complex 3D human neuroarchitecture. In contrast, next-generation microphysiological platforms demonstrate significantly superior predictive validity; for example, microfluidic organ-on-a-chip and neuromuscular 3D systems have successfully recapitulated human-specific TDP-43 aggregation, axonal dismantling, and therapeutic rescue profiles (such as combined rapamycin and bosutinib efficacy) that closely mirror clinical responses. To overcome this translational bottleneck, the most promising priority development direction lies in the technological convergence of patient-derived 3D organoids/assembloids integrated within automated microfluidic organ-on-a-chip devices and 3D bioprinted biofunctional hydrogels. Coupled with CRISPR-Cas9 isogenic control generation and AI-driven multi-omics readouts, this hybrid paradigm establishes perfusable, physiologically relevant human motor neuraxis models capable of high-content drug screening and early biomarker validation. In Table 4, a critical comparison between traditional and advanced experimental platforms in the modelling of ALS is reported.

Finally, it is important to underline that the ultimate molecular truth of ALS involves post-mortem brain banking and cell/liquid biopsy, permitting us to understand the cellular biology and variability of neurodegenerative diseases, which can also directly accelerate ALS breakthroughs. Post-mortem brain banking is essential for providing ground-truth human tissue histopathology, validating TDP-43/FUS/SOD1 staging, and confirming whether targets identified in 3D iPSC models reflect late-stage human disease end-states [121]. Serial sampling of patient biofluids (plasma/CSF) for extracellular vesicles, neurofilament light chain (NfL), and circulating microRNAs enables early presymptomatic detection and real-time monitoring of therapeutic response when coupled with patient-derived 3D cell models [122].

In conclusion, the true future of ALS precision medicine lies in integrated datasets linking the knowledge acquired by next-generation cell culture models with longitudinal biofluid data collected during a patient’s life and the cellular, genomic, and spatial pathology of donated tissue after death. This cross-scale approach allows artificial intelligence to trace a peripheral blood signal back to specific molecular failures in the motor cortex, accelerating both drug discovery and early diagnosis.

## Figures and Tables

**Table 1:** Overview, core applications, pros, and cons of 2D cell culture models in ALS research.

Model/System Type	Setup/Description	Core Applications in ALS	Key Advantages (Pros)	Major Limitations (Cons)	REF
Immortalized cell lines	Monolayer cultures grown in dispersed 2D format.	Protein localization (SOD1, TDP-43, FUS). Oxidative stress and autophagy assays. Preliminary chemical screening.	High accessibility and low cost. Ease of culture and maintenance. Simple genetic manipulation	Low physiological CNS relevance. Lack of patient cellular diversity. Risk of immortalization artefacts	[24,25]
Patient-derived somatic cells (fibroblasts and lymphoblasts)	Primary non-neuronal cells from patient skin/blood biopsies	Donor material for iPSC reprogramming. Peripheral biomarker profiling. Subtype metabolic profiling	Easily accessible patient tissue. Retains patient genetic background. Enables longitudinal donor tracking	Non-neuronal lineage. Cannot model motor neurodegeneration. Lacks synaptic/neural machinery	[28]
Primary human CNS cells	Glial and neural cells obtained from post-mortem CNS tissues	Cell-type specific vulnerability. Native neuron–glia metabolic crosstalk. Target validation in human CNS	High biological fidelity. Native human CNS microenvironment. Validates late-stage disease state	Extreme tissue scarcity. Cannot maintain long-term in culture. Cannot model presymptomatic stages. High operational cost	[26,27]
iPSC-derived monocultures (neurons and glia)	Reprogrammed patient cells differentiated into specific neural/glial lineages.	Modelling fALS and sALS mechanisms. Cell-autonomous toxicity discovery. Isogenic drug testing (CRISPR-Cas9)	Retains patient’s exact genetic background. Human-specific disease fidelity. Compatible with CRISPR isogenic controls	High culture maintenance cost. Variable reprogramming efficiency. Lack of standardized differentiation. Absence of ageing signatures	[28,29]
2D co-culture systems (Direct/Transwell)	Multicellular co-cultures (MNs + glia/muscle) direct or via porous membranes.	Non-cell-autonomous toxicity. Glia-mediated neuroinflammation. Early NMJ signalling breakdown	Replicates cell–cell communication. Captures soluble neurotoxic factors. Models multi-lineage interaction	Lacks 3D spatial tissue architecture. Altered morphology and cell death kinetics. Inability to model fluidic circulation	[30]

**Table 2:** Key characteristics, distinct roles, pros, cons, and solutions for ameliorating application of next-generation 3D cell models for ALS research.

3D Model	Main Description	Distinct Roles and ALS Applications	Key Advantages (Pros)	Major Limitations (Cons)	Potential Solutions
**Organoids** **(Cortical/Cerebral)**	iPSC-derived 3D cerebral organoids with regionalized cortical layer architecture	Modelling upper MN and cortical network degeneration. Recapitulating early C9orf72 poly-GA DPR toxicity and synaptic loss. Drug screening targeting UPR/PERK pathways (e.g., GSK2606414)	Extended neuronal survival (>12 months). Retains donor genetic background. Captures complex cortical circuit dynamics	Core necrosis from vascularization deficits. Lack of innate microglial population. Line-to-line differentiation variability	Air–liquid interface (ALI) slice cultures. Co-culture with iPSC-derived microglia. Integration with microfluidic perfusion
**Organoids** **(Spinal Cord)**	Ventrally patterned 3D spinal organoids containing lower motor neurons and glia	Modelling ventral horn lower motor neuron degeneration. Dissecting C9orf72 loss-of-function pathological mechanisms. Screening pro-inflammatory cytokines (IL-6, TNFα) in 3D	Replicates human spinal neurogenesis. Forms central canal-like neural tube structures. Amplifies neuroinflammatory signalling	Absence of systemic circulatory flow. High batch-to-batch structural heterogeneity. Limited long-term axonal extension	Guided morphogen gradient protocols. Bioprinted hydrogel scaffold alignment. Microfluidic spinal-on-a-chip embedding
**Organoids** **(Neuromuscular)**	3D co-differentiation of motor neurons, skeletal muscle fibres, and Schwann cells	Dissecting distal axonal denervation and “dying-back” mechanisms. Evaluating muscle contractility, weakness, and NMJ failure. Testing therapeutic reversal of C9orf72/TDP-43 NMJ deficits	Recapitulates functional, contractile NMJs. Captures early peripheral synaptic breakdown. Enables functional electrophysiological readouts	High technical culture complexity. Muscle fibre maturation constraints. Operational handling and force sensing challenges	Flexible pillar force-sensing chips. Optogenetic motor neuron stimulation. Automated multi-well microfluidics
**Chimeroids**	Multi-donor chimeric 3D organoids generated by pooling neural progenitors from multiple individuals	Evaluating patient-specific genetic susceptibility across multiple donors. Direct side-by-side comparison of fALS/sALS mutations in identical environment. Eliminating batch-to-batch operational noise in drug screening	Overcomes culture-to-culture batch variability. Avoids clonal dominance of fast-growing lines. High-content multi-donor screening capacity	Complex single-cell donor lineage tracking. Maturation stage standardization needs. Vascularization limits in large structures	Single-cell RNA sequencing profiling. Refined chimeric culture media formulations. Integration with microfluidic chips
**Assembloids** **(Cortico-Motor)**	3D multi-region structures formed by physical fusion of cortical, spinal, and muscle spheroids	Reconstructing the functional 3D human “motor neuraxis”. Modelling long-range corticospinal axonal projections and circuit breakdown. Examining anterograde “dying-forward” vs. “dying-back” disease spreading	Recreates multi-organ connectivity in vitro. Demonstrates light/cortical-driven muscle contraction. Enables dissection of circuit-wide dysfunction	Lacks resident immune cells if unsupplemented. High technical assembly failure rate. Complex long-term culture maintenance	Incorporation of microglial spheroids. Standardization of fusion timing and media. Microfluidic compartment alignment
**Assembloids** **(Neuroimmune)**	Fusion of neural/cortical organoids with iPSC-derived microglial spheroids or macrophages	Modelling glia-mediated non-cell-autonomous toxicity. Investigating neuroinflammatory propagation and synaptic pruning. Target discovery for microglial activation pathways (e.g., C9orf72, FUS)	Restores native immune surveillance in 3D. Captures inflammatory cytokine crosstalk. High relevance for non-cell-autonomous toxicity	Risk of uncontrolled microglial activation. Difficulty tuning physiological glia ratios. Variable microglial migration depth	Controlled microglial seeding ratios. Cytokine/chemokine gradient optimization. Real-time imaging of microglial dynamics
**Organ-on-a-Chip** **Microfluidics**	Microfluidic platforms with compartmentalized microgrooves and dynamic fluid flow	Modelling axonal transport, NMJ denervation, and BBB impairment under flow. Establishing precise pharmacological and therapeutic drug gradients (e.g., rapamycin) High-content drug testing (e.g., rapamycin + bosutinib combination)	Continuous CSF/blood-like fluidic perfusion. Precise localized chemical gradient generation. Compatible with MEA electrophysiology and biosensors	Hydrophobic PDMS drug absorption. Fluid shear stress and altered gas exchange. High fabrication and manual setup complexity	Transition to thermoplastic plates (PS/COC). Automated, micro-moulded multi-chip plates. Surface anti-absorption coatings

**Table 3:** Summary of in vivo animal models engineered via CRISPR/Cas9 genomic editing strategy for the study of ALS.

Organism	Target Gene	Editing Strategy	Key Pathological Insights	REF.
***Danio rerio (Zebrafish)***	VCP	Constitutive Knockout	Model establishment for VCP dynamics	[88]
	TP73	Knockout	Impaired motor neuron development, abnormal axonal structure	[89]
	TDP-43	Loss-of-Function	Establishment of loss-of-function phenotypes	[90]
***Drosophila melanogaster***	VCP	Knock-in	Disease mechanisms in multisystem proteinopathy/ALS	[91]
***Caenorhabditis elegans***	FUS	Knock-in	Autophagy impairment driving neuromuscular defects	[92]
	TDP-43	Conditional Depletion	DNA double-strand break repair defects and altered DDR signalling	[93]
	SOD-1	Endogenous Knock-in	Recapitulation of neurotransmitter-type specificity	[94]
**Rat**	FUS	Knock-in (R521C)	Physiological role of FUS in sleep, circadian rhythm, and learning	[95]
**Mouse**	MATR3	Knock-in (S85C)	Model mimicking early-stage ALS for basic and preclinical research	[96]
	CREST (SS18L1)	Knockout/Knock-in (Q394X)	Neuroinflammatory responses and ALS-like motor defects	[97]
	TDP-43	Knock-in (M337V, G298S)	Establishment of loss-of-function phenotypes	[98]
	C9orf72	Knockout/Deficiency	Model for hexanucleotide repeat expansion associated with FTLD/ALS	[99,100]

**Table 4:** Critical comparison between traditional and advanced experimental systems for modelling ALS.

Parameter	2D Cell Cultures	In Vivo Animal Models	Organoids/Assembloids	Microfluidics Systems
**Genetic origin**	Immortalized cell lines or primary murine cells	Transgenic	Human iPSCs	Human iPSCs
**Tissue architecture**	Two-dimensional monolayer; absence of 3D polarity and extracellular ECM	Three-dimensional, complex and systemic (whole organism)	Three-dimensional; self-organized into cortical-like layers	Three-dimensional or pseudo-3D; compartmentalized and geometrically defined
**Cellular Heterogeneity**	Low (monocultures or simple co-cultures)	Very high (immune system, glia, neurons, vessels)	Medium–high (neurons, astrocytes, oligodendrocytes; microglia often absent if not integrated)	Controlled (precise co-cultures of selected cell types)
**Blood–Brain Barrier**	Not present	Present	Absent (unless endothelial co-differentiation)	Highly reproducible (BBB-on-a-chip models)
**Mechanical Forces/Flow**	Absent (static conditions)	Present (physiological blood flow and cerebrospinal fluid)	Absent (static conditions or limited orbital agitation)	Present (controlled microfluidic flow, generated shear stress)
**Ageing Modelling**	Not possible	Partial (accelerated in rodents, does not reflect human senescence)	Difficult (fetal/juvenile epigenetic identity; requires chemical induction)	Partial (focused on acute responses or short-term induced stress)
**High-Throughput Screening**	Excellent	Very low (expensive, slow, ethical limitations)	Medium (limited by batch-to-batch variability and costs)	Medium–high
**Cost and time**	Low; rapid results (days)	High; long times (months/years)	High; long maturation times (months)	Medium–high; technical setup times, but rapid experimental runs
**Main Limitations**	Artefacts; minimal biological relevance to brain complexity	Very low clinical translation	Core necrosis (lack of vascularization); high heterogeneity	Simplified macroscopic architecture; reduced long-term autonomy
**Predictive Validity**	Very Low (<10%) fails to model complex non-cell-autonomous toxicity or 3D pathology	Low (<10–15%) captures motor symptoms but lacks human pathology and human kinetics	High (70–85%) recapitulates early human synaptic loss and UPR pathways	Very High (80–90%) precisely recapitulates human NMJ dismantling and axonal transport loss
**Clinical Translation**	Minimal; prone to high false-positive and false-negative drug rates	Poor (<5–10% clinical success); over 90–95% of rodent-validated drugs fail in Phase II/III trials	High; validates human-specific therapeutic targets	Very High; accurately predicts human clinical dosage and synergistic efficacy
**High-Throughput Screening Capacity**	Excellent; rapid automated screening	Very Low; limited by ethical rules (3Rs), high costs, and slow readouts	Medium; limited by batch variability and long maturation	Medium–High; transitioning to standardized, automated multi-chip microfluidic plates

## Data Availability

No new data were created or analyzed in this study. Data sharing is not applicable to this article.

## Abbreviations

The following abbreviations are used in this manuscript:

ALS	Amyotrophic Lateral Sclerosis
CNS	Central Nervous System
fALS	Familial Amyotrophic Lateral Sclerosis
sALS	Sporadic Amyotrophic Lateral Sclerosis
SOD1	Superoxide Dismutase-1
C9ORF72	Chromosome 9 Open Reading Frame 72
TDP-43	TAR DNA-Binding Protein 43 kDa
FUS	Fused In Sarcoma
HREs	Hexanucleotide Repeat Expansions
GWAS	Genome-Wide Association Study
WES	Whole-Exome Sequencing
FDA	U.S. Food and Drug Administration
iPSCs	Induced Pluripotent Stem Cells
PBMCs	Peripheral Blood Mononuclear Cells
NMJ	Neuromuscular Junction
NMs	Motor Neurons
ECM	Extracellular Matrix
EBs	Embryonic Bodies
DPR	Poly-GA Dipeptide Repeat
UPR	Unfolded Protein Response
PERK	Protein kinase R-like Endoplasmic Reticulum Kinase
WT	Wild Type
BMP	Bone Morphogenetic Protein
Shh	Sonic Hedgehog
qRT-PCR	Quantitative Real-Time PCR
RNA-seq	RNA Sequencing
CSF	Cerebrospinal Fluid
BBB	Blood–Brain Barrier
CRISPR-Cas9	Clustered Regularly Interspaced Short Palindromic Repeats-associated system 9
VCP	Valosin-Containing Protein
MATR3	Matrin-3
CREST	Calcium-Responsive Transactivator
